# A CYPome-wide study reveals new potential players in the pathogenesis of Parkinson’s disease

**DOI:** 10.3389/fphar.2022.1094265

**Published:** 2023-01-19

**Authors:** Philip Hartz, Tobias Fehlmann, Gudrun Wagenpfeil, Marcus Michael Unger, Rita Bernhardt

**Affiliations:** ^1^ Institut für Biochemie, Fachbereich Biologie, Universität des Saarlandes, Naturwissenschaftlich-Technische Fakultät, Saarbrücken, Germany; ^2^ Institut für Klinische Bioinformatik, Universität des Saarlandes, Saarbrücken, Germany; ^3^ Institut für Medizinische Biometrie, Epidemiologie und Medizinische Informatik, Universität des Saarlandes, Homburg, Germany; ^4^ KLinik für Neurologie, Fachbereich Klinische Medizin, Universität des Saarlandes, Homburg, Germany; ^5^ Klinik für Neurologie, SHG Kliniken Sonnenberg, Saarbrücken, Germany

**Keywords:** cytochrome P450, Parkinson’s disease, CYP46A1, POR, Adx, xenobiotica, cholesterol, PPMI

## Abstract

Genetic and environmental factors lead to the manifestation of Parkinson’s disease (PD) but related mechanisms are only rudimentarily understood. Cytochromes P450 (P450s) are involved in the biotransformation of toxic compounds and in many physiological processes and thus predestinated to be involved in PD. However, so far only SNPs (single nucleotide polymorphisms) in *CYP2D6* and *CYP2E1* have been associated with the susceptibility of PD. Our aim was to evaluate the role of all 57 human P450s and their redox partners for the etiology and pathophysiology of PD and to identify novel potential players which may lead to the identification of new biomarkers and to a causative treatment of PD. The PPMI (Parkinson’s Progression Markers Initiative) database was used to extract the gene sequences of all 57 P450s and their three redox partners to analyze the association of SNPs with the occurrence of PD. Applying statistical analyses of the data, corresponding odds ratios (OR) and confidence intervals (CI) were calculated. We identified SNPs significantly over-represented in patients with a genetic predisposition for PD (GPD patients) or in idiopathic PD (IPD patients) compared to HC (healthy controls). Xenobiotic-metabolizing P450s show a significant accumulation of SNPs in PD patients compared with HC supporting the role of toxic compounds in the pathogenesis of PD. Moreover, SNPs with high OR values (>5) in P450s catalyzing the degradation of cholesterol (CYP46A1, CY7B1, CYP39A1) indicate a prominent role of cholesterol metabolism in the brain for PD risk. Finally, P450s participating in the metabolism of eicosanoids show a strong over-representation of SNPs in PD patients underlining the effect of inflammation on the pathogenesis of PD. Also, the redox partners of P450 show SNPs with OR > 5 in PD patients. Taken together, we demonstrate that SNPs in 26 out of 57 P450s are at least 5-fold over-represented in PD patients suggesting these P450s as new potential players in the pathogenesis of PD. For the first time exceptionally high OR values (up to 12.9) were found. This will lead to deeper insight into the origin and development of PD and may be applied to develop novel strategies for a causative treatment of this disease.

## Introduction

Parkinson’s disease (PD) is the second most common neurodegenerative disorder. It was shown that the number of people with PD doubled to over six million from 1990 to 2015 and is further increasing (Dorsey et al., 2018). The main characteristic for PD is the degeneration of the *Substantia nigra pars compacta* and the connected loss of dopaminergic neurons. However, so far due to lack in understanding the reason of this degeneration, causative treatment is not possible. Therefore, intense research is going on trying to decipher the pathogenesis of PD, to find new biomarkers for early detection and novel candidates and approaches for treatment. While the majority of PD cases are idiopathic (IPD), about 5%–10% are familial and linked to mutations in several genes (GPD) (Bandres-Ciga et al., 2020). Mutations in more than 20 genes have been associated with this disease during the past years and 90 more were found in a recent genome-wide association study (Nalls et al., 2019). On the other hand, it is assumed that in the case of IPD the disease is caused and modulated by a complex interplay of genetic and environmental factors (Kalia and Lang, 2015).

A starting point for our investigations were reports on a higher susceptibility of patients for PD in the case of activity-decreasing mutations of the cytochrome P450 (P450) CYP2D6. P450s are hemo-proteins and in humans they are involved in the biotransformation of drugs, the conversion and bioactivation as well as degradation of environmental substances as well as the biosynthesis of endogenous compounds like steroid hormones, prostaglandins, vitamin D and others (Bernhardt, 2021). P450s are localized on the cytoplasmic side of the endoplasmic reticulum and on the luminal side of the inner mitochondrial membrane. They are monooxygenases and obtain electrons from NAD(P)H *via* a short redox chain, a NADPH-dependent FAD- and FMN-containing reductase (POR) in case of microsomal P450s and an iron-sulfur protein of the [2Fe-2S] type, adrenodoxin (Adx), and a FAD-containing reductase, adrenodoxin reductase (AdR) in case of mitochondrial P450s (Omura, 2010). In humans, 50 out of 57 P450s are located in the endoplasmic reticulum, while seven are mitochondrial (Omura, 2006). P450s have been found in many organs, including the brain (Ghosh et al., 2016). Much less is known on their specific functions in the brain. CYP2D6, an important drug metabolizer (Tyndale et al., 1991; Rendic and Guengerich, 2015), as well as CYP2E1 (Vaglini et al., 2004) were found to convert the neurotoxin 1-methyl-4-phenyl-1,2,3,6-tetrahydropyridine (MPTP) which was shown to induce PD-like symptoms in mouse models of PD. Later, a correlation between the incidence of PD and the occurrence of so-called poor metabolizers (PMs) was described (Smith et al., 1992; Deng et al., 2004; Elbaz et al., 2004; Anwarullah et al., 2017). However, the results are conflicting (Riedl et al., 1998; Nicholl et al., 1999; Ur Rasheed et al., 2017) and need further research. A pooled odds ratio (OR) from ten published studies of 1.47 was calculated for PMs for PD susceptibility by (McCann et al., 1997).

We used the well-accepted PPMI (The Parkinson’s Progression Markers Initiative) database to analyze possible relations between mutations in the genes of selected proteins and the origin and development of PD. The association of individual SNPs in the genes of all 57 P450s as well as their three redox partners (POR, Adx, AdR) with PD was analyzed. We observed statistically significant changes in patients suffering from IPD *versus* healthy controls (HC) and in patients with a genetic predisposition (mutations of the *LRRK2, SNCA, PRKN* and other genes) for PD (GPD) *versus* HC and identified several *P450s* displaying a significant over-representation of selected SNPs in PD patients compared with the HC.

## Materials and methods

### Extraction of the data on 57 P450s, POR, Adx and AdR from the PPMI database

Lists for the variants were obtained by downloading the whole-genome sequencing (WGS) VCF files of the Parkinson’s Progression Markers Initiative (PPMI; July 2018 release). The PPMI study is an ongoing international multicenter cohort study to identify biomarkers of progression in PD (Marek et al., 2018). Briefly, these were generated based on whole-blood extracted DNA samples and processed with the Centers for Common Disease Genomics standard pipeline, with the human hg38 assembly as reference genome (Regier et al., 2018). Subsequently, we annotated the called variants with SnpEff 5.0 (Cingolani, et al., 2012b) and SnpSift 5.0 (Cingolani et al., 2012a). Variants were further processed with the R programming language and aggregated per diagnosis group at the allele level. An overview of the workflow is given in Supplementary Figure S1.

Besides genomic data, information on group assignment (HC, IPD, GPD) for the investigated subjects were extracted from the PPMI database (https://ida.loni.usc.edu/login.jsp?project=ppmi). Genomic data as well as information on group assignment were merged into one data set for analyses.

### Statistical analysis of the obtained data

Data analysis was performed using IBM-SPSS Version 26. Any *p* values reported are two-sided and subject to a significance level of 0.05. Due to the explorative nature of the study, we did not account for the issue of multiple statistical testing. Thus, we report raw 2-sided *p*-values without adjustment. Risk factors were identified with logistic regression and reported as odds ratios (OR) with 95% confidence intervals (CI).

## Results

### Bioinformatic approach

We extracted the variants from the whole-genome sequencing data of the PPMI cohort for the 57 cytochromes of the P450 family as well as their three redox partners. We thereby obtained allele information for 40,514 genomic positions, of which 3,256 were located in protein coding regions. The potential effect of the SNPs was forecasted using the available programs SnpEff and SnpSift, which combine data on functional consequences of SNPs from the GWAS Catalog, SIFT, Mutation Taster as well as ClinVar (Supplementary Figure S1). Most of the found SNPs are new and have been classified as “modifiers” of the corresponding gene function by the programs used. It has to be mentioned that in few cases the SNPs in individual *P450s* (or the three redox partners) were not found with highest frequency in the reference genome but in the studied individuals from the PPMI database. This might be due either to mistakes in the assignment of the corresponding SNP in the reference genome or to adaptation leading to an over-representation of the corresponding SNP in the studied PPMI cohort. Where necessary, we will mention this point for individual SNPs.

### SNPs in the genes of human P450 systems and Parkinson’s disease

To get an overview on the *P450* species, which display an over-representation of SNPs in their genes in PD patients, we categorized them into three groups: HC, IPD, GPD. Data from 193 HC representing the control group, 362 IPD and 317 GPD, representing the two classes of patients, were extracted from the PPMI database. Based on this data, we performed statistical analyses to find out how many SNPs in the genes encoding the investigated 60 proteins are significantly different between IPD and HC or GPD and HC. Since we considered only one gene at a time in these three groups, we did not perform corrections for multiple testing to get a direct impression about the occurrence of certain SNPs in different individuals. As a starting point, we focused on the association between certain SNPs and the occurrence of IPD or GPD. OR values showing the corresponding over- or under-representation of a certain SNP in a gene were calculated. Only data where the statistically obtained probability values (*p*-values) were below 0.05 were taken into account and are discussed in this paper. When the OR value is >1, an over-representation (higher odds) of the corresponding SNP occurs in PD patients. If OR is < 1, then the corresponding SNP is under-represented (lower odds) in PD patients and over-represented in HC this way suggesting a protective effect. Since we identified for some *P450s* many different SNPs with very high or high statistical significance, we grouped the data into five main groups: SNPs with OR values > 5, showing a significant over-representation of the SNP in PD patients, SNPs with over-representation between 2 and 5, groups with OR values between 0.5 and 2.0 demonstrating a medium effect, SNPs with more than 5-fold over-representation in HC (OR<0.2) and SNPs with 2-5-fold over-representation in HC (OR between 0.2 and 0.5). As demonstrated in Tables 1, 2, only two P450s, *CYP1A1* and *CYP17A1*, did not show any SNPs which were significantly different between PD patients and HC although a total of 86 and 99 SNPs was found in *CYP1A1* and *CYP17A1*, respectively. Four P450s (*CYPs 1A2, 7A1, 11B1, 27B1*) displayed differences with OR values of 0.5–2.0. Intriguingly, 26 P450s showed SNPs with OR values > 5. It is of importance to mention that most SNPs with OR values > 5 (and also between 2 and 5) are found in GPD patients suggesting that they may potentiate the effect of a genetic predisposition caused by known PD-inducing mutations to a considerable extent. Only SNPs in *CYP46A1, Adx* and *POR* were found in IPD patients with OR values > 5, while SNPs in 20 out of the 56 remaining *P450s* displayed OR values between two and five in IPD patients. The identification of so many significant SNPs in *P450s* was completely unexpected but suggests a significant contribution of these enzymes to the pathogenesis or development of PD. It is also of interest that the two immediate redox partners of P450s (*Adx, POR*) contain SNPs which were found > 5-fold over-represented in PD patients compared with HC. *POR* also shows two SNPs with OR<0.2 in IPD and one in GPD patients while *Adx* has three SNPs with OR<0.2 in GPD patients. Moreover, 21 and 34 *P450s* display SNPs with OR values < 0.2 in IPD and GPD patients (Table 2), respectively, with *CYP51A1* and *CYP4B1* having the highest numbers of SNPs with OR <0.2. SNPs with OR values < 0.2 indicate a protective effect on the pathogenesis of PD. In the following paragraphs we discuss the putative association of SNPs in different *P450s* with PD for the various classes of P450s as divided according to their major substrate class by (Guengerich, 2017).

**TABLE 1:** Different effects of SNPs of various CYPs and redox partners on Parkinson’s disease.

CYPs without any effect	1A1, 17A1
CYPs and redox partners with SNPs with OR < 0.2	1B1, 2A6, 2A7, 2A13, 2C8, 2C19, 2E1, 2F1, 2R1, 2U1, 2W1, 3A4, 3A5, 3A7, 3A43, 4A11, 4B1, 4F3, 4F8, 4F11, 4F12, 4F22, 4V2, 4X1, 4Z1, 5A1, 7B1, 8A1, 8B1, 19A1, 20A1, 24A1, 27A1, 27C1, 39A1, 46A1, 51A1, Adx, POR
CYPs with OR < 0.2 and no ORs >5	1B1, 2R1, 2U1, 2W1, 3A4, 3A5, 3A7, 3A43, 4A11, 4F8, 4F11, 4F22, 4X1, 8B1, 19A1, 24A1, 27A1, 27C1, 51A1
CYPs with marginal effects (OR = 0.50–1.99)	1A2, 7A1, 11B1, 27B1
CYPs and redox partners with SNPs between OR 2.00–5.00 but no OR > 5.00	1B1, 2J2, 2U1, 3A4, 3A5, 3A43, 4A11, 4F2, 4F11, 4F22, 4X1, 11A1, 11B2, 19A1, 21A2, 24A1, 27C1, 51A1, AdR
CYPs and redox partners with SNPs between OR 2.00–5.00 and with OR > 5.00	2A6, 2B6, 2C8, 2C9, 2C19, 2D6, 2E1, 2F1, 2S1, 4B1, 4F3, 4F12, 4V2, 4Z1, 5A1, 7B1, 8A1, 20A1, 39A1, 46A1, Adx, POR
CYPs and redox partners with SNPs with OR > 5.00	2A6, 2A7, 2A13, 2B6, 2C8, 2C9, 2C18, 2C19, 2D6, 2E1, 2F1, 2S1, 4B1, 4F3, 4F12, 4V2, 4Z1, 5A1, 7B1, 8A1, 20A1, 26A1, 26B1, 26C1, 39A1, 46A1, Adx, POR
CYPs with OR > 5.0 and no OR < 0.2	2B6, 2C9, 2C18, 2D6, 2S1, 26A1, 26B1, 26C1

**TABLE 2:** Summary of significant SNPs in 57 cytochrome P450s and three redox partners. *CYP*: cytochrome P450 gene; GPD and IPD display the numbers of significant SNPs found in patients suffering from GPD or IPD, respectively; number of total SNPs shows the number of all SNPs (significant and non-significant) found in the corresponding gene. Significant means with *p* < 0.01 or 0.05. The data are subdivided according to the function of the individual SNPs as described by Guengerich (Guengerich, 2017).

Substrate class	*CYP*	Total SNPs	Significant SNPs			OR < 0.2		OR = 0.2–0.5		0.5 <OR <2.0		OR = 2.0–5.0		OR > 5	
			**GPD**	**IPD**	**SUM**	**GPD**	**IPD**	**GPD**	**IPD**	**GPD**	**IPD**	**GPD**	**IPD**	**GPD**	**IPD**
Drugs	*1A1*	86	−	−	−	−	−	−	−	−	−	−	−	−	−
	*1A2*	130	1	−	1	−	−	−	−	1	−	−	−	−	−
	*2A6*	231	24	9	33	6	1	11	−	2	8	3	−	2	−
	*2A13*	188	4	1	5	1	1	−	−	−	−	−	−	3	−
	*2B6*	584	107	7	114	−	−	1	−	69	7	36	−	1	−
	*2C8*	591	51	88	139	−	2	2	1	23	47	25	38	1	−
	*2C9*	981	33	3	36	−	−	1	−	28	1	3	2	1	−
	*2C18*	1935	9	1	10	−	−	3	1	5	−	−	−	1	−
	*2C19*	2908	30	4	34	−	2	6	2	12	−	4	−	8	−
	*2D6*	153	11	2	13	−	−	1	2	3	−	4	−	3	−
	*2E1*	965	18	96	114	3	1	6	14	2	4	6	77	1	−
	*2F1*	336	12	3	15	2	−	1	1	1	1	2	1	6	−
	*3A4*	331	4	−	4	2	−	1	−	−	−	1	−	−	−
	*3A5*	424	6	7	13	1	2	4	3	−	2	1	−	−	−
	*3A7*	387	4	−	4	1	−	2	−	1	−	−	−	−	−
	*3A43*	548	13	29	42	3	1	6	21	1	6	3	1	−	−
Fatty Acids	*2J2*	436	27	1	28	−	−	16	−	9	1	2	−	−	−
	*2U1*	334	6	16	22	2	−	1	1	2	14	1	1	−	−
	*4A11*	238	12	−	12	1	−	2	−	−	−	9	−	−	−
	*4B1*	1218	72	18	90	20	4	21	6	22	3	5	5	4	−
	*4F11*	547	141	25	166	2	1	39	17	100	6	−	1	−	−
	*4F12*	313	6	10	16	−	1	4	6	1	1	−	2	1	−
	*4F22*	877	29	16	45	1	1	3	1	16	11	9	3	−	−
	*4V2*	510	51	4	55	1	1	10	1	37	2	2	−	1	−
Eicosanoids	*4F2*	409	11	12	23	−	−	3	4	8	6	−	2	−	−
	*4F3*	653	55	16	71	4	−	14	1	20	9	16	6	1	−
	*4F8*	313	58	73	131	2	1	3	32	53	40	−	−	−	−
	*5A1*	4394	205	134	339	16	26	77	40	54	56	48	12	10	−
	*8A1*	1074	19	5	24	1	1	8	3	3	1	6	−	1	−
Vitamins	*2R1*	184	10	2	12	3	−	1	2	6	−	−	−	−	−
	*24A1*	424	43	10	53	2	−	6	3	15	6	20	1	−	−
	*26A1*	123	1	1	2	−	−	−	1	−	−	−	−	1	−
	*26B1*	366	13	2	15	−	−	6	−	6	2	−	−	1	−
	*26C1*	269	1	3	4	−	−	−	1	−	2	−	−	1	−
	*27B1*	79	4	−	4	−	−	−	−	4	−	−	−	−	−
	*27C1*	754	45	3	48	1	−	20	3	21	−	3	−	−	−
Sterols	*1B1*	977	17	47	64	7	−	3	5	2	36	5	6	−	−
	*7A1*	157	1	−	1	−	−	−	−	1	−	−	−	−	−
	*7B1*	2849	23	10	33	5	−	7	4	1	3	3	3	7	−
	*8B1*	54	7	−	7	2	−	−	−	5	−	−	−	−	−
	*11A1*	413	9	−	9	−	−	−	−	1	−	8	−	−	−
	*11B1*	205	1	19	20	−	−	1	−	−	19	−	−	−	−
	*11B2*	212	2	6	8	−	−	−	−	1	6	1	−	−	−
	*17A1*	99	−	−	−	−	−	−	−	−	−	−	−	−	−
	*19A1*	2022	43	7	50	2	2	9	2	30	3	2	−	−	−
	*21A2*	102	14	3	17	−	−	7	1	−	1	7	1	−	−
	*27A1*	475	7	5	12	2	2	5	2	−	1	−	−	−	−
	*39A1*	1700	191	10	201	3	−	24	3	89	3	74	4	1	−
	*46A1*	734	9	4	13	1	−	3	−	1	1	1	1	3	2
	*51A1*	986	157	12	169	4	8	2	2	70	2	81	−	−	−
Unknown	*2A7*	246	48	4	52	1	−	4	2	37	2	−	−	6	−
	*2S1*	279	8	8	16	−	−	2	1	3	6	1	1	2	−
	*2W1*	173	15	−	15	1	−	9	−	5	−	−	−	−	−
	*4A22*	233	37	−	37	−	−	22	−	15	−	−	−	−	−
	*4X1*	385	36	2	38	1	−	31	2	3	−	1	−	−	−
	*4Z1*	781	133	2	135	2	1	119	−	8	1	2	−	2	−
	*20A1*	1011	57	6	63	2	3	7	2	8	−	38	1	2	−
Redox Partner	*AdR*	183	5	−	5	−	−	3	−	1	−	1	−	−	−
	*Adx*	667	28	6	34	3	−	22	3	2	1	1	−	−	2
	*POR*	1797	98	29	127	1	2	23	19	48	4	17	3	9	1

### Association of SNPs in the genes of various *P450s* with PD

Due to the large quantity of obtained data (Table 2), we will focus in this paper mainly on SNPs in genes which display OR>5 or <0.2 and discuss possible effects on the derived P450 function.

#### CYP2D6 and CYP2E1

Based on initial reports on an increased occurrence of PD in CYP2D6 PMs as well as a higher incidence of activity-decreasing SNPs in *CYP2E1* of PD patients (McCann et al., 1997; Vaglini et al., 2004), we first analyzed the distribution of SNPs in these two genes in PD patients and HC. In *CYP2D6*, 13 out of 153 total SNPs were found to be statistically significantly different compared to HC, 11 in GPD and two in IPD patients (Table 2).

Three SNPs in the gene of *CYP2D6* were found with OR values of 9.54, 9.54 and 9.49 in patients suffering from GPD (compared to HC) (Table 2; Table 3) demonstrating that GPD patients have an up to 9.5-fold over-representation (higher odds) of one of these gene variations compared with HC. This means that only patients which have a predisposition for PD due to mutations in one of the genes like *LRRK2, SNCA etc.* are prone to develop PD when the *CYP2D6* gene is mutated, whereas none of the SNPs alone (OR values in IPD patients of 0.228 and 0.229 were found) seems to be sufficient to cause symptoms of PD. SNP rs76015180 (OR = 9.49) was found in intron 5, while the other two SNPs with OR values > 5 are in the downstream region. The functional effect for the corresponding *CYP2D6* gene products was classified as “modifier” by the used programs and no other information was found in the literature with regard to these SNPs.

**TABLE 3:** Summary of selected single nucleotide polymorphisms (SNP) with statistically significant association (*p*-value <0.05) to Parkinson´s Disease (PD) for various P450s. The strength of association is represented by the odds ratio (OR) for patients with genetic PD (GPD) or idiopathic PD (IPD) and the healthy control group (HC), respectively. Only SNPs discussed in the paper are shown. The confidence interval (CI) of the OR is indicated by the lower and upper limit for a confidence level of 95%.

Gene	SNP	OR	95% CI		*p*-value	Comparison
			Lower	Upper		
*CYP2A6*	rs373949046 ^a^	6.453	1.491	27.921	0.013	GPD/HC
	rs28399461 ^a^	5.659	1.689	18.957	0.005	GPD/HC
*CYP2A7*	rs3815706 ^b^	9.310	1.210	71.641	0.032	GPD/HC
	rs4079370 ^b^	9.310	1.210	71.641	0.032	GPD/HC
*CYP2A13*	chr19:41089804 ^a^	9.717	1.270	74.338	0.028	GPD/HC
	rs114493299 ^a^	11.559	1.531	87.290	0.018	GPD/HC
*CYP2B6*	rs113314890 ^c^	5.152	1.579	16.810	0.007	GPD/HC
*CYP2C8*	rs11572151 ^a^	8.871	1.157	68.007	0.036	GPD/HC
*CYP2C9*	rs191947947 ^a^	5.076	1.154	22.325	0.032	GPD/HC
*CYP2C18*	chr10:94735937 ^a^	5.076	1.154	22.325	0.032	GPD/HC
*CYP2C19*	chr10:94855423 ^a^	8.238	1.069	63.477	0.043	GPD/HC
	chr10:94827399 ^a^	8.238	1.069	63.477	0.043	GPD/HC
*CYP2D6*	rs28371725 ^b^	0.229	0.06	0.899	0.035	IPD/HC
	rs28371706 ^a^	0.228	0.07	0.752	0.015	IPD/HC
	rs142302759 ^a^	9.536	1.25	72.779	0.03	GPD/HC
	rs184086520 ^a^	9.536	1.25	72.779	0.03	GPD/HC
	rs76015180 ^a^	9.487	1.24	72.402	0.03	GPD/HC
*CYP2E1*	rs2070676 ^b^	3.914	1.158	13.223	0.028	IPD/HC
	rs2070676 ^a^	3.739	1.069	13.083	0.039	IPD/HC
	chr10:133540620 ^a^	8.211	1.066	63.268	0.043	GPD/HC
*CYP2F1*	chr19:41114592 ^a^	11.559	1.531	87.290	0.018	GPD/HC
	rs115159306 ^a^	11.559	1.531	87.290	0.018	GPD/HC
*CYP2S1*	chr19:41193298 ^a^	8.871	1.157	68.007	0.036	GPD/HC
	chr19:41205398 ^a^	8.289	1.075	63.901	0.042	GPD/HC
*CYP4B1*	rs144531409 ^a^	12.242	1.625	92.193	0.015	GPD/HC
	rs180941912 ^a^	8.871	1.157	68.007	0.036	GPD/HC
*CYP4F3*	rs28371541 ^a^	10.206	1.343	77.583	0.025	GPD/HC
*CYP4F12*	rs190731214 ^c^	8.434	1.098	64.804	0.040	GPD/HC
*CYP4V2*	chr4:186209599 ^a^	5.412	1.236	23.687	0.025	GPD/HC
*CYP4Z1*	chr1:47103933 ^a^	12.929	1.721	97.130	0.013	GPD/HC
	chr1:47071196 ^a^	9.536	1.250	72.779	0.030	GPD/HC
*CYP5A1*	rs182750452 ^a^	10.880	1.436	82.420	0.021	GPD/HC
	rs188670782 ^a^	10.880	1.436	82.420	0.021	GPD/HC
	chr7:139800028 ^a^	10.206	1.343	77.583	0.025	GPD/HC
	chr7:139866119 ^a^	10.206	1.343	77.583	0.025	GPD/HC
*CYP7B1*	rs16931331 ^b^	6.365	1.451	27.912	0.014	GPD/HC
	rs16931331 ^b^	4.433	1.003	19.586	0.049	IPD/HC
	rs16931334 ^b^	6.365	1.451	27.912	0.014	GPD/HC
	rs16931334 ^b^	4.433	1.003	19.586	0.049	IPD/HC
	rs146085563 ^a^	5.897	1.764	19.710	0.004	GPD/HC
	rs191573334 ^a^	5.659	1.689	18.957	0.005	GPD/HC
	rs148614983 ^a^	5.659	1.689	18.957	0.005	GPD/HC
	rs142000295 ^a^	5.659	1.689	18.957	0.005	GPD/HC
	chr8:64621935 ^a^	5.076	1.154	22.325	0.032	GPD/HC
*CYP8A1*	rs117140207 ^a^	8.211	1.066	63.268	0.043	GPD/HC
*CYP20A1*	chr2:203251819 ^a^	8.984	2.112	38.222	0.003	GPD/HC
	rs55939578 ^b^	6.151	1.392	27.183	0.017	GPD/HC
*CYP26A1*	chr10:93070649 ^a^	10.206	1.343	77.583	0.025	GPD/HC
*CYP26B1*	rs187119740 ^a^	6.089	1.402	26.437	0.016	GPD/HC
*CYP26C1*	rs202086264 ^a^	10.206	1.343	77.583	0.025	GPD/HC
*CYP39A1*	chr6:46605698 ^a^	8.901	1.161	68.233	0.035	GPD/HC
*CYP46A1*	rs4905883 ^a^	10.946	1.235	97.022	0.032	IPD/HC
	rs4905883 ^b^	9.272	1.073	80.084	0.043	IPD/HC
	rs113274473 ^a^	10.206	1.343	77.583	0.025	GPD/HC
	chr14:99722542 ^a^	9.536	1.25	72.779	0.03	GPD/HC
	chr14:99686510 ^a^	8.871	1.157	68.007	0.036	GPD/HC
	rs139952469 ^b^	0.114	0.014	0.914	0.041	GPD/HC
*Adx*	rs117805978 ^a^	8.904	1.172	67.661	0.035	IPD/HC
	rs146768862 ^a^	8.879	1.168	67.465	0.035	IPD/HC
*POR*	rs190398380 ^a^	8.871	1.157	68.007	0.036	GPD/HC
	chr7:75983340 ^b^	8.613	0.998	74.324	0.050	GPD/HC

^a^ Heterozygous allele combination (wildtype | SNP).

^b^ Homozygous allele combination (SNP | SNP).

^c^ Population of low frequent homozygous SNP, combinations that are differing from the main SNP.

In *CYP2E1* a total of 965 SNPs was identified in patients and HC from the data base, 114 of them being statistically different among patients and controls (Table 2). Only one SNP (chr10133540620) had an OR value > 5 (OR = 8.21) and was found in GPD patients, while three SNPs were found with OR = 0.15 in GPD patients and one SNP with OR = 0.10 is present in IPD patients suggesting a protective role of these latter SNPs. These five SNPs are located in a regulatory genomic position and described as modifiers of the function. The high number of SNPs with OR values between two and five in IPD patients (Table 2) indicates that CYP2E1 might have a more pronounced effect on the development of PD than CYP2D6. In a recent study on PD progression also changes in the CYP2E1 gene expression have been described (Kern et al., 2021). This might be due to its prominent role in the conversion of xenobiotics (acetaminophen, anesthetics), alcohols, acrylamide, carcinogens and halogenated alkanes (Guengerich, 2020). Interestingly, the SNP with OR>5 belonged to a group of SNPs which were not most frequently found in the reference gene but in homozygous patients (see above). Among the 77 SNPs over-represented in IPD patients, SNP rs2070676 (OR = 3.74 and 3.91 in heterozygous and homozygous form, respectively) which is located in intron 7, was before shown to be associated with PD (Shahabi et al., 2009). Interestingly, these authors also observed a susceptibility for PD by the C-allele being much more prominent than the G-allele (as in our case), but only in patients without relatives having PD (corresponding to IPD). It may be speculated that SNPs in *CYP2E1* could affect ROS production or its capability to detoxify potential neurotoxins this way promoting the development of PD and supporting the importance of environmental factors for the risk to develop PD.

#### Association of SNPs in other xenobiotic-metabolizing P450s with PD

After supporting the role of the xenobiotic-metabolizing P450s CYP2D6 and CYP2E1 for the development of PD and identifying new SNPs being over-represented in GDP and IPD patients, we decided to look at the other 14 xenobiotic-metabolizing P450s (Guengerich, 2017) to find out whether other ones play a prominent role in PD. Interestingly, CYP1A1 and CYP1A2 are not or barely related to PD (Table 1; Table 2), although a total of 86 and 130 SNPs was observed in *CYP1A1* and *CYP1A2*, respectively. When it comes to the members of the CYP2 family involved in the metabolism of xenobiotics, it turns out that all of them display SNPs with OR values >5 in case of GPD patients. This again suggests the importance of toxic compounds and due to this to environmental factors on the development of PD. In addition to this, (besides *CYP2E1*, which is discussed above) *CYP2A6, CYP2B6, CYP2C8, CYP2C9, CYP2C19 and CYP2F1* show SNPs which are 2–5 times over-represented in GPD patients, while SNPs with similar OR in *CYP2C8, CYP2C9* and *CYP2F1* genes were found to be over-represented in IPD patients. In contrast to this, members of the CYP3A family do not show such a strong variation in their genes in PD patients compared to HC. None of them (*CYP3A4, CYP3A5, CYP3A7, CYP3A43*) displays a SNP with an OR value > 5 in PD patients. When looking at SNPs with OR values < 0.2 it becomes clear that seven out of 15 *P450* genes (*CYP2D6* and *2E1* are being discussed in the previous paragraph) display one or two of these protecting SNPs with OR<0.2 in IPD patients and eight in GPD patients.

Taken together, our data indicates the importance of environmental toxins for the risk to develop PD and on a close relationship between endogenous (genetic) and exogenous (environmental) factors for the pathogenesis of this disease.

#### Analysis of SNPs in P450s involved in fatty acid conversion

As shown in Table 2, only three *P450* genes (*CYP4B1, CYP4F12 and CYP4V2*) contain SNPs with OR values > 5 in GPD patients but no such SNP was found in IPD patients. However, five of eight *P450s* display OR values between two and five in IPD patients and six of eight in GPD patients. This demonstrates that fatty acids also seem to be of importance for the development of IPD. When taking a closer look onto the substrates of the most prominent P450s of this group, it turns out that the predominant reaction catalyzed by members of the CYP4 family is the ω-hydroxylation of fatty acids and eicosanoids which leads to a removal of excess free fatty acids and the production of bioactive metabolites like 20-HETE (20-hydroxyeicosatetraenoic acid) synthesized from arachidonic acid (Hsu et al., 2007). CYP4B1 seems to be involved in the production of 12-HETE (12-hydroxy-5,8,11,14-eicosatetraenoic acid) (Mastyugin et al., 1999). In addition, its participation in endogenous as well as xenobiotic metabolism has been described (Thesseling et al., 2020) and it was demonstrated to catalyze the production of cytotoxic metabolites (Kowalski et al., 2019). When looking at protective SNPs, it turns out that *CYP4B1* shows the highest numbers with four SNPs having OR values < 0.2 in IPD and 20 in GPD patients. This is not surprising when taking into account that in the genes of this *P450* a total of 1218 SNPs was found, whereas this number varied between 238 (*CYP4A11*) and 877 (*CYP4F22*) for the other *CYP4* genes (Table 2).

#### Analysis of SNPs in P450s involved in eicosanoid metabolism

Eicosanoids are generated from arachidonic acid and are oxidized derivatives of this and other polyunsaturated fatty acids (PUFAs). Eicosanoids play a pivotal role in inflammation and immunity. Besides cyclooxygenase and lipoxygenase, P450s play an essential role in their biosynthesis (Calder, 2020). CYP8A1 catalyzes the isomerization of prostaglandin H_2_ to prostacyclin (Wada et al., 2004), while CYP5A1 converts the cyclooxygenase product prostaglandin H_2_ to thromboxane A_2_ (Hsu et al., 1999). *CYP5A1* is highly polymorphic. With 4.394 SNPs found in the individuals of the PPMI database it shows the highest number of SNPs in a *P450*. Out of the 4.394 SNPs 339 are significantly different between PD patients and HC. Four SNPs were found with OR values > 10 in GPD patients (Table 3). All of them are attributed to regulatory genomic positions. Also, in *CYP8A1* and *CYP4F3* SNPs with OR values > 5 have been described in GPD patients. *CYP8A1* displays one SNP with OR = 8.21 in GPD patients in the regulatory region, described as modifier (Table 3). The SNP in *CYP4F3* with OR = 10.21 comprises also a variation in a regulatory genomic position suggested to be a modifier. It has been found that CYP4F3 is a leukotriene B_4_ ω-hydroxylase which leads to a powerful proinflammatory mediator (Kikuta et al., 2002). This observation indicates that P450s involved in eicosanoid metabolism are of special importance for the development and progression of PD in patients, which have already a predisposition due to another PD-relevant mutation in the genome and thus may play an outstanding role in the pathogenesis of PD.

#### Analysis of SNPs in P450s involved in vitamin metabolism

Seven P450s (CYPs 2R1, 24A1, 26A1, 26B1, 26C1, 27B1, 27C1) were shown to be involved in the metabolism of vitamins (Guengerich, 2017). Only three of them (all being members of the CYP26 family) show one SNP each with an OR value > 5 (between 6 and 10) in GPD patients (Table 3). All three members of the CYP26 family are involved in retinoic acid (RA) metabolism (Guengerich, 2017) thus indicating a possible effect of retinoids on the development of PD. This coincides well with previous observations that retinoic acid is involved in neuronal differentiation (Janesick et al., 2015). In addition, it was very recently discussed that altered vitamin A metabolism and bioavailability contributes to oxidative stress, neuroinflammation and disturbance in biological rhythms indicating on multiple ways how retinoic acid may be associated with the pathogenesis of PD (Marie et al., 2021). Also, CYP27C1 is involved in the desaturation of retinoids (Glass et al., 2021), but demonstrates only a rather moderate association with PD with only three SNPs with OR values between two and five in GPD patients. However, one SNP was found with OR<0.2 in GPD patients suggesting a protecting effect. Interestingly, SNPs for genes involved in the biosynthesis and degradation of vitamin D (*CYP2R1, CYP27B1, CYP24A*) (Schuster, 2011) do not correlate to a high extent (OR values > 5 or <0.2) with the occurrence of PD. This indicates that the biosynthesis of vitamin D is obviously not related in a very significant manner to PD, but rather may have a moderate effect.

#### Analysis of SNPs in sterol-metabolizing and steroid-synthesizing P450s

The 14 P450s shown in Table 2 in the sterol section can be divided into those being involved in steroid hormone biosynthesis (CYP11A1, CYP11B1, CYP11B2, CYP17A1, CYP19A1, CYP21A2), those involved in the conversion of cholesterol and oxy-cholesterol (CYP7A1, CYP7B1, CYP27A1, CYP39A1, CYP46A1) and those involved in the metabolism of sterols like estradiol and bile acids (CYP1B1, CYP8B1) (Lorbek et al., 2012; Niwa et al., 2015; Guengerich, 2017). A special case is CYP51A1 catalyzing the removal of the C14 methyl group of lanosterol leading to cholesterol, which is the evolutionary most conserved P450 enzyme and is expressed ubiquitously. As shown in Table 2, P450s catalyzing steroid hormone biosynthesis (reactions reviewed in (Bernhardt and Neunzig, 2021)) demonstrate no or only moderate association to PD. In contrast, among the P450s involved in oxy-sterol metabolism*, CYP7B1, CYP39A1* and *CYP46A1* have SNPs with OR>5 in GPD patients (Table 2; Table 3). CYP46A1 is located specifically in the central nervous system and catalyzes the conversion of cholesterol to 24OH-cholesterol this way promoting its excretion from the brain (Petrov and Pikuleva, 2019). As shown, *CYP46A1* displayed 734 total SNPs in the investigated PPMI cohort and is the only *P450* that shows SNPs with OR >5 in IPD patients (Table 2; Table 3). However, the corresponding base in the reference group is not the most frequent one. In addition to this, three SNPs with OR>5 (being 10.21, 9.54 and 8.87) were discovered in GPD patients. The three SNPs are located in regulatory regions and described as modifiers. One SNP is found with an OR = 0.11 in GPD patients and is located in a regulatory genomic position. This data demonstrates a very significant association between SNPs in *CYP46A1* and the occurrence of PD with one mutation being effective without predisposition and others enhancing the negative effect of the predisposition to a significant extent. CYP7B1, being a 7α-hydroxylase of 24OH-cholesterol, shows seven SNPs with OR>5 which are located in regulatory genomic positions and classified as modifiers. The SNP in the *CYP39A1* gene is a variant located in a regulatory region as well. Interestingly, SNPs rs16931331 and rs16931334 in *CYP7B1* show OR values of 6.36 in GPD patients and 4.43 in IPD patients thus underlining their special impact on the pathogenesis of PD. In contrast, CYP7A1, described to be a cholesterol 7α-hydroxylase (Wu et al., 1999), is not significantly associated with PD, while CYP27A1 being a human sterol 27-hydroxylase (Ali et al., 2013) displays several SNPs with OR<0.2 indicating a more protective effect on the development of PD. CYP8B1, which like CYP1B1 is involved in the metabolism of sterols, also does not display a strong association with the occurrence of PD. Finally, *CYP51A1* demonstrates no SNPs with OR>5, but has 81 SNPs with OR values between two and five in GPD. This suggests that out of the 169 SNPs present in the corresponding gene, about half of them is over-represented in PD. Together this data indicates a very important association of sterol, especially cholesterol metabolism, and the pathogenesis of PD.

#### Analysis of SNPs in P450s with so far unidentified function (orphan P450s)

Seven P450s have been classified as P450s with unknown function by Guengerich (Guengerich, 2017), but for some of them substrates have been described more recently although detailed studies are still missing. CYP2A7 shows 97% sequence identity in the exons with CYP2A6 and a connection to nicotine metabolism has been found (Gao et al., 2020). A role of CYP2S1 in the ω-1 hydroxylation of polyunsaturated fatty acids has been described in an untargeted metabolomic study (Fekry et al., 2019). The enzyme is induced by dioxin and seems to play also an important role in *in-situ* toxicity, primarily in extra-hepatic tissues. It is highly expressed in the brain (Yan et al., 2018). CYP2W1 is expressed in a cancer-specific way and converts pro-dugs as well as fatty acids like arachidonic acid (Pan and Ong, 2017). CYP20A1 is highly expressed in the brain, especially in the *Substantia nigra* (Stark et al., 2008; Lemaire et al., 2016)*,* and a connection to methotrexate toxicity has been found (Papapetropoulos et al., 2004; Aslibekyan et al., 2014) although this finding is controversial (Koponen et al., 2022). Interestingly, for CYPs of the CYP4 family (CYP4A22, CYP4X1 and CYP4Z1) a participation in fatty acid conversion and epoxide formation of eicosatrienoic acids has been described (Zöllner et al., 2009; Carver et al., 2014; McDonald et al., 2017; Durairaj et al., 2019). When looking at the association of SNPs in these genes (CYPs with unidentified function) with PD, it becomes clear that the largest effects owing six SNPs with OR values > 5 in GPD patients are identified in *CYP2A7* (Table 2). Five of these SNPs were found to be homozygous and also five are intron variants with a described modifier effect. One homozygous SNP was found as a missense mutation leading to an amino acid exchange Met204Val. No information on the functional consequences of this amino acid replacement has been described in the literature so far. Also, *CYP2S1, CYP4Z1* and *CYP20A1* have two SNPs each with OR values > 5 in GPD patients. Most of them are located in regulatory regions and described by our bioinformatic tool as having a moderate impact. It needs to be mentioned that *CYP4Z1* demonstrates 781 total SNPs in our PPMI cohort with only very few being significantly higher in PD patients compared to HC. The two SNPs discussed here have OR values of 9.54 and 12.93 suggesting that they are significantly over-represented in PD. Also, *CYP20A1* shows many total SNPs (1011) but only 2 with OR>5. Both are described as variants in regulatory genomic positions and modifiers. *CYP4A22*, which so far has not been found in the brain, does not show any SNPs with OR values ≥ 2. Together this indicates on a special importance of CYP2A7, CYP20A1, CYP2S1 and CYP4Z1 for the pathogenesis of PD.

### Association of SNPs in the genes of P450 redox partners with PD

#### POR

As most of the human P450s, POR is located on the cytoplasmic side of the endoplasmic reticulum. We found one SNP associated with IPD (with OR = 6.86) and nine associated with GPD displaying OR values of >5 (Table 2). This indicates that changes in POR also seem to be strongly associated with the occurrence of PD. When looking at the genome position of the SNP with OR>5 in IPD patients, it turns out that it is located in a regulatory genomic position of heterozygous patients. The same SNP is also found in different heterozygous GPD patients with an OR value between 2.84 and 5.36 as well as in heterozygous IPD patients with OR values between 3.08 and 3.27. Bioinformatic analyses described this SNP as modifier of activity. However, the reference genome does not show the most frequent base at that place (see above). Unfortunately, no more information is available concerning the effect of this SNP on the expression and activity of POR. Some significant SNPs in GPD patients seem to be clustered in the regulatory region with five SNPs displaying exactly the same OR of 8.57 and two with OR = 8.61 and 8.87, respectively. The SNPs with OR = 8.57 and 8.61 again do not display the “original” base in the reference gene. In contrast, SNP rs190398380 (OR = 8.87) which is also described as being located in the regulatory region shows the base defined as “original” one in the reference gene. When looking at the SNPs, which show a strong protective effect on PD (OR<0.2), it becomes clear that two of them can be found in IPD patients and one in GPD patients. All three coincide with the reference gene. The two SNPs found in the genome of IPD patients with OR<0.2 are homozygous and described to be located in regulatory genomic positions. To both SNPs a modifier function has been prescribed. The SNP with OR = 0.197 in GPD patients is also located in a regulatory region and described as modifier. Interestingly, SNP rs1057868 (also known as POR*28) has not been found as a significant SNP in our PD patient cohort.

#### Adx

Adx (assigned as Fdx1 in the database) is the immediate electron transfer protein to mitochondrial P450s. Interestingly, two SNPs were found with an OR value > 5 (8.90 and 8.88) in IPD patients (Table 2). These two SNPs are heterozygous, located in intron regions and described as modifiers. In GPD patients three protective SNPs with OR values of 0.11, 0.15 and 0.20 were found. All of them are intron variants and also described as modifiers. This data indicates that Adx may have a significant effect on the pathogenesis of PD, especially since high OR values have been obtained for IPD patients.

#### AdR

AdR (assigned as FDXR in the database) transfers the electrons from NADPH in two one-electron steps to Adx. No SNPs were found displaying statistically significant differences between IPD patients and HC, but one heterozygous SNP displayed an OR value of 2.24 in GPD patients (Table 2). This indicates a low or moderate effect of AdR on the etiology of PD.

## Discussion

Although PD is the second most frequent neurodegenerative disease world-wide, its etiology is still unknown. To create novel approaches for the causative treatment of this disease, new biomarkers and targets have to be identified and characterized. Based on early studies on the interrelation between polymorphisms in the *CYP2D6* gene, leading to decreased elimination of neurotoxins, and the occurrence of PD, we aimed to investigate the contribution of all 57 members of the P450 superfamily to the development of PD. Members of the P450 family play a prominent role not only in the detoxification and bioactivation of xenobiotics, but also in endogenous pathways like steroid hormone metabolism, biosynthesis of fatty acids, vitamins and eicosanoids *etc.* And they are, therefore, involved in many physiological and pathophysiological processes (Bernhardt, 2021).

Surprisingly, their contribution to the risk of developing PD has barely been studied so far (Breckenridge et al., 2016) although besides CYP2D6 15 more P450s are involved in the biotransformation of drugs and xenobiotics so that it is challenging to investigate their potential as a risk factor for PD. Furthermore, the effect of P450-catalyzed endogenous reactions on the pathogenesis of PD has not been investigated so far.

**FIGURE 1:**
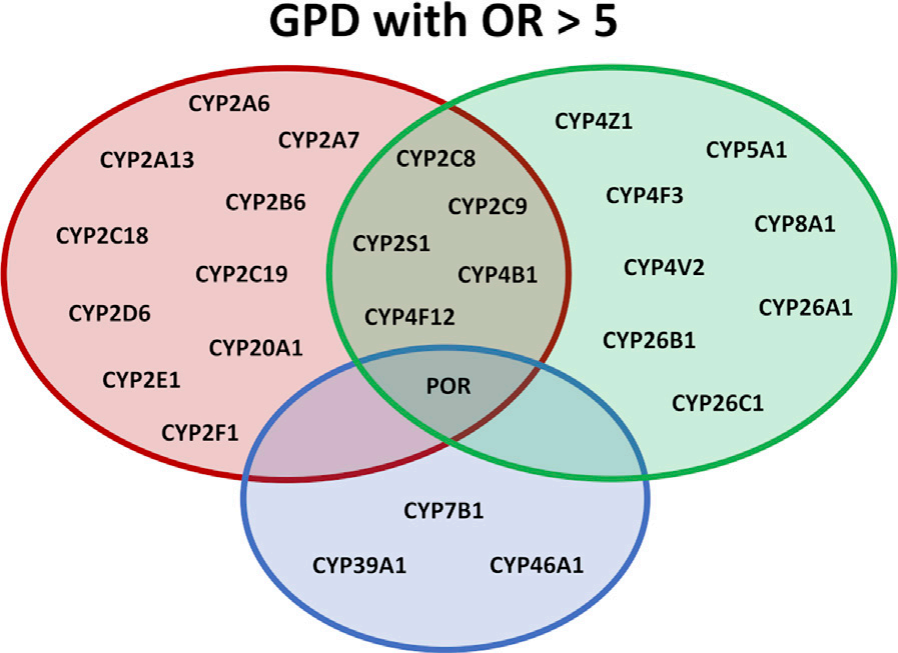
Overview of the effect of different SNPs in cytochromes P450 on various physiological pathways. P450s are shown which display OR values >5.0 in GPD patients. In the red circle P450s are listed participating in the biotransformation of xenobiotica, in the green circle P450s are listed participating in immune response and inflammation and in the blue circle P450s are shown involved in sterol metabolism or the degradation of cholesterol.

Our data clearly indicate a significant contribution of xenobiotic-converting P450s as a cause or promoting factor of PD and for the first time identify important SNPs in *P450s* involved in drug (and xenobiotic) metabolism associated with PD. As shown, all P450s belonging to the *CYP2* family display an over-representation of SNPs in the genes of GPD patients with OR values > 5. Eight of these 10 P450s are described for the first time as linked to PD (Table 1; Table 2; Table 3 and Figure 1). CYP2C19 and CYP2F1 seem to play a prominent role for the pathology of GPD with eight and six SNPs displaying OR values > 5, respectively. In contrast, CYP1A1 and CYP1A2 are of no or low importance, while *CYP3A7* has three significant SNPs, however, with OR values <0.5 suggesting a protective role against PD. Our data on CYP2D6 also show a clear and significant over-representation of several SNPs in the *CYP2D6* gene of patients suffering from GPD with OR values of up to 9.5 (Table 3). This result is especially remarkable when taking into account that previous studies on a correlation of SNPs in so-called “poor metabolizers” with PD reported an average OR value of 1.47 (McCann et al., 1997), which is more than 6-fold lower than the value we found in our studies. Although the OR values for these SNPs is very high in GPD patients, no association of *CYP2D6* with PD-causing genes has been found in GWAS studies (Edwards et al., 2010; Li et al., 2022). However, associations between CYP2D6 and pesticides on PD risk have been described in some studies and are also found between LRRK2 und PINK1 and pesticides (Dardiotis et al., 2013). Moreover, our data may shed light onto the partially conflicting results obtained when studying the correlation of PD with the occurrence of the “poor metabolizer” type. While the previously published studies did not differentiate between GPD and IPD patients, our investigations, taking this fact into account, clearly demonstrate that only GPD patients have a significant over-representation of several SNPs compared with HC, while in IPD patients only two under-represented SNPs have been found suggesting a protective effect of these SNPs. This data may explain why the various studies in the literature came to inconsistent findings. It seems that when the amount of GPD patients in the studied cohort is high, a correlation with PD becomes detectable, while it can barely be observed when the portion of GPD patients is too low to be statistically relevant. The bioinformatic tools used in our study classified the observed SNPs in *CYP2D6* mostly as modifiers. Literature data concerning their function are not available. Experimental investigations, which are outside the scope of this study are necessary and certainly will contribute to a deeper understanding of the relation between the observed SNPs and the manifestation of PD. This is particularly interesting, since CYP2D6 has also been shown to catalyze the formation of dopamine from p-tyramine (Hiroi et al., 1998). This suggests that a lower expression or activity of CYP2D6 could also reduce the production of dopamine *via* this pathway. It is of special interest that SNPs in *CYP2E1* and *CYP2C8* also exert a strong influence on the development of IPD. While a connection between CYP2E1 and PD has been described in previous studies (Shahabi et al., 2009; Kern et al., 2021), the contribution of CYP2C8 as a risk factor for IPD is completely new. The effect of CYP2E1 can be mainly traced to ROS production, whereas the effect of CYP2C8 SNPs on the metabolism is not clear yet. However, it has been shown that CYP2C8 expression can be increased in human brain cells by the organophosphorous pesticide moncrotophos and that exposure to this pesticide on the other hand causes an increase in ROS production leading to oxidative damage (Tripathi et al., 2017).

Taken together, the data found here demonstrate an association between PD and SNPs in drug-metabolizing and xenobiotic-converting enzymes. This strongly supports the hypothesis that toxic substances may have a devasting effect on the initiation and/or development of PD underlining the importance of environment-gene interaction as a significant factor for the pathology of PD (Figure 1).

The second group of *P450s* displaying SNPs with high OR values is connected to fatty acid metabolism, especially biosynthesis and metabolism of eicosanoids (Figure 1). As can be seen from Table 2 and Table 3, especially *CYP5A1* displays a high variability of the gene with 10 SNPs having OR values > 5 and 4 with OR>10. Moreover, besides CYP5A1, which catalyzes the biosynthesis of thromboxane A_2_, CYP8A1, a prostacyclin synthase, shows a SNP with an OR value of 8.21. Both products, thromboxane and prostacyclin, are involved in inflammatory processes. Another P450, CYP4F3, catalyzes the production of leukotriene B4, a strong proinflammatory mediator, whereas CYP4F12, CYP4V2 and CYP4Z1 participate in fatty acid hydroxylation which can lead to the biosynthesis of bioactive HETE metabolites also playing a role in inflammation (Zöllner et al., 2009; Carver et al., 2014; McDonald et al., 2017; Durairaj et al., 2019). Furthermore, CYP4B1, which, besides participating in the formation of 12-HETE, is also involved in the metabolism of xenobiotics, displays four SNPs that are 6.67–12.24 over-represented in GPD patients compared to HC. In addition to CYP4B1 also CYPs 2C8, 2C9, 2S1 and 4F12 are involved in both, xenobiotic as well as fatty acid/eicosanoid metabolism, thus being on the interface of both metabolic pathways and possibly affecting the development of PD from both sides (Figure 1). This indicates for the first time that SNPs in 19 *P450s* involved in the biosynthesis of fatty acids and eicosanoids are closely linked to PD, even in cases where no predisposition by mutations of known PD causing genes exists (Table 2) and underlines the outstanding importance of eicosanoids and of inflammation (Sarparast et al., 2020) for neurodegenerative diseases and as a risk factor of PD (Figure 1).

In addition to P450s participating in the conversion of toxic substances and the production of immune modulators, a third group of *P450s* shows high numbers of SNPs with significant OR values. They belong to the group of sterol-converting enzymes. Here, CYP46A1, which is highly expressed in various regions of the brain (Petrov and Pikuleva, 2019; Pikuleva and Cartier, 2021), plays an outstanding role (Table 2; Figure 1). It is the only *P450* with a SNP with OR>5 (10.94 and 9.27 in heterozygous and homozygous patients, respectively) found in IPD patients and three SNPs with OR>5 in GPD patients. As discussed above, it has, however, to be taken into account that the SNP in IPD patients as well as in three of the four SNPs with OR values < 0.2 show the most frequent base in the SNP and not in the reference genome. Interestingly, the occurrence of Alzheimer’s disease was also related to a SNP in one of the introns of *CYP46A1*. An OR value of 1.69, about 5-fold lower than for the association of some of the SNPs with PD found here, has been described for this disease (He et al., 2012). Besides *CYP46A1*, also *CYP7B1* displays OR values > 5 in GPD patients as does *CYP39A1*. The three P450s are involved in the conversion of cholesterol. While CYP46A1 catalyzes the conversion of cholesterol to 24OH-cholesterol, CYP39A1 converts this substance to a 7α product to finally synthesize bile acids. Interestingly, an increase in the level of 24OH-cholesterol has been observed in the cerebrospinal fluid of PD patients (Björkhem et al., 2013; Björkhem et al., 2018). CYP7B1 is also highly expressed in the brain and a potent oxysterol 7α-hydroxylase with 27OH-cholesterol as main substrate, which is formed by CYP27A1. This data suggests that the metabolism, especially the degradation, of cholesterol in the brain is of pivotal importance for the pathogenesis of PD. This conclusion is further supported when looking at SNPs in IPD patients with OR values between two and five displaying one, four and three SNPs in *CYP46A1, CYP39A1* and *CYP7B*1, respectively.

Since P450s are monooxygenases, they need electrons to perform oxygen activation and the following substrate hydroxylation. Mutations in the redox proteins may cause varying effects on different P450s (Ewen et al., 2011; Pandey and Flück, 2013). With SNPs displaying OR values > 5 in IPD patients, *POR* and *Adx* are significantly linked to PD. This is supported by the finding that for *POR* nine SNPs with OR>5 are found in GPD patients. The SNPs found are located in regulatory regions and no detailed information about their influence on the activities of POR or Adx with various P450s is available.

There are also some limitations of the investigations presented here. First of all, the data do not allow individual characterization of the two alleles of a studied person. Secondly, our analyses considered individual SNPs but did not analyze the multitude of various combinations of SNPs from different *P450s* in individual patients, since this was outside the focus of this paper, is very time-consuming and requires additional independent investigation. Finally, although suggestions concerning the functional consequences of the observed SNPs were drawn, experimental studies are missing. This is, however, common to most of the identified PD risk factors so that only very few mechanistic details are available to explain the pathogenesis of PD. Analysis of the data obtained in our studies reveal that most of the 40,514 SNPs in *P450s* were found in regulatory genomic positions (introns, UTR) and only 3,256 are located in exons. This is not surprising when taking into account that only 1%–2% of the human genome represent protein-coding structures and that 22,000 genes produce a proteome with over 200,000 distinct proteins (Annalora et al., 2017). It supports the importance of non-coding regions for the regulation of physiological processes and the pathogenesis of many diseases as well as the identification of new targets for drug treatment (Eisenstein, 2021).

Summarizing our findings, the data demonstrate for the first time a significant contribution of cytochrome P450 systems for the development of PD. CYP46A1 as well as the direct redox partners of P450s, POR and Adx, have been found to possess SNPs in IPD and GPD patients with OR>5. This is a very strong association, especially when taking into account that previous associations between SNPs of CYP2D6 (one of the only two studied P450s concerning their possible contribution to the occurrence of PD) and PD had a mean OR of 1.47, more than 6-fold lower than observed in the present paper. The strongly affected P450s were classified into three main groups concerning their function (Figure 1): (i) P450s involved in the biotransformation of xenobiotics, (ii) P450s involved in fatty acid and eicosanoid metabolism and (iii) P450s participating in the degradation of cholesterol. Our data for the first time give detailed information on enzymes, especially P450s and their redox partners, contributing to the development of PD by three mechanisms: toxicity, inflammation, and cholesterol degradation. We thus were able to identify novel potential players involved in the pathogenesis of this disease, but extensive and time-consuming experimental studies are needed to get insight into mechanisms of PD development, especially since most of the SNPs are located in regulatory regions.

## Data Availability

The original contributions presented in the study are included in the article/Supplementary Materials, further inquiries can be directed to the corresponding authors.

## Abbreviations

CYP, cytochrome P450; P450, cytochrome P450; Adx, adrenodoxin; POR, NADPH-dependent cytochrome P450 reductase; CYP, P450; Adx; AdR and POR designates thecorresponding genes of P450, Adx, AdR and POR; PD, Parkinson’s disease; IPD, idiopathic (sporadic) PD; GPD, PD with a genetic predisposition; PPMI, The Parkinson’s Progression Markers Initiative; OR, odds ratio; CI, confidence interval.
