# Supplementary material for: A CYPome-wide study reveals new potential players in the pathogenesis of Parkinson’s disease
Source: Front Pharmacol. 2023 Jan 19;13:1094265. doi: 10.3389/fphar.2022.1094265 (PMC9892771; doi:10.3389/fphar.2022.1094265)
Supplement: Supplementary file 1 [file DataSheet1.docx]

**Supplementary Data**


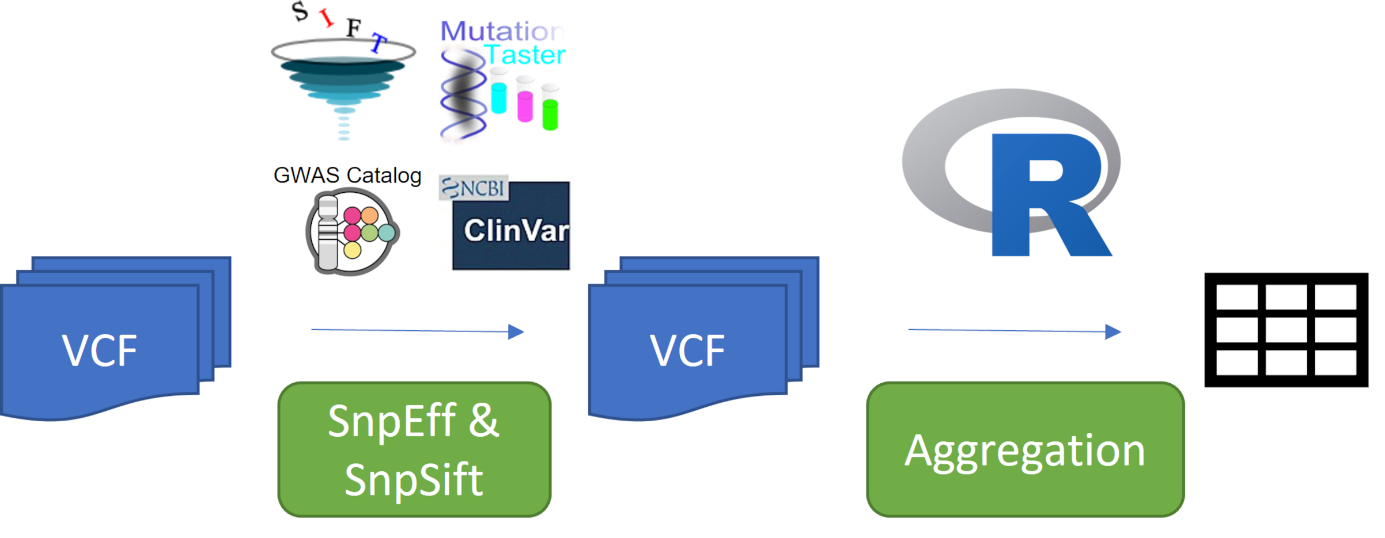


**Fig. S1.** Workflow for the extraction and processing of the data from the PPMI database.
